# Metagenomic Analysis of Five Phylogenetically Distant Anammox Bacterial Enrichment Cultures

**DOI:** 10.1264/jsme2.ME22017

**Published:** 2022-07-09

**Authors:** Mamoru Oshiki, Yoshihiro Takaki, Miho Hirai, Takuro Nunoura, Atsushi Kamigaito, Satoshi Okabe

**Affiliations:** 1 Division of Environmental Engineering, Faculty of Engineering, Hokkaido University, Sapporo, Japan; 2 Super-cutting-edge Grand and Advanced Research (SUGAR) Program, Institute for Extra-cutting-edge Science and Technology Avant-garde Research (X-STAR), Japan Agency for Marine-Earth Science and Technology (JAMSTEC), 2–15 Natsushima-cho, Yokosuka 237–0061, Japan; 3 Research Center for Bioscience and Nanoscience (CeBN), JAMSTEC, 2–15 Natsushima-cho, Yokosuka 237–0061, Japan

**Keywords:** anammox bacteria, metagenomic ana­lysis, *Chloroflexota*, *Bacteroidota*

## Abstract

Anaerobic ammonium-oxidizing (anammox) bacteria are slow-growing and fastidious bacteria, and limited numbers of enrichment cultures have been established. A metagenomic ana­lysis of our 5 established anammox bacterial enrichment cultures was performed in the present study. Fourteen high-quality metagenome-assembled genomes (MAGs) were obtained, including those of 5 anammox *Planctomycetota* (*Candidatus* Brocadia, *Ca.* Kuenenia, *Ca.* Jettenia, and *Ca.* Scalindua), 4 *Bacteroidota*, and 3 *Chloroflexota*. Based on the gene sets of metabolic pathways involved in the degradation of polymeric substances found in *Chloroflexota* and *Bacteroidota* MAGs, they are expected to be scavengers of extracellular polymeric substances and cell debris.

The anaerobic ammonium oxidation (anammox) process in which NH_4_^+^ is oxidized to N_2_ gas with NO_2_^–^ markedly contributes to the global nitrogen cycle (Kuypers *et al.*, 2005), and has been installed in full-scale wastewater treatment plants as a cost-efficient and environmentally-friendly nitrogen removal process (van der Star *et al.*, 2007; Ali and Okabe, 2015). Anammox bacteria were discovered in the mid-1990s, and belong to a deep-branching monophyletic group tentatively proposed in the order *Brocadiales* of the bacterial phylum *Planctomycetota* (Strous *et al.*, 1999). The following five candidate genera have been identified in the order: *Ca.* Brocadia, *Ca.* Kuenenia, *Ca.* Jettenia, *Ca.* Anammoxoglobus, and *Ca.* Scalindua. Although many researchers have attempted to enrich and isolate anammox bacteria, a pure culture has not yet been obtained. This is somewhat surprising because anammox bacteria may be highly enriched in membrane bioreactors (<98% in total biomass) (Lotti *et al.*, 2014), and a subsequent buoyant density separation technique enables the further enrichment of anammox bacteria (>99.9%) (Strous *et al.*, 1999; Kartal *et al.*, 2011). Therefore, several reasons for unsuccessful isolation attempts have been proposed, such as the occurrence of cell density-dependent anammox activity (Strous *et al.*, 1999; Oshiki *et al.*, 2020; Zhang and Okabe, 2020) and microbial interactions between anammox bacteria and coexisting microorganisms; *e.g.*, symbiotic/cooccurring bacteria supply the micronutrients required for the growth of anammox bacteria (Kindaichi *et al.*, 2004; Mee *et al.*, 2014; Kim *et al.*, 2021). Although the micronutrients required for the growth of anammox bacteria have not yet been identified, the supply of soluble organic matter from anammox bacteria was shown to support the growth of heterotrophs in an anammox bacterial enrichment culture (Ni *et al.*, 2012). A previous metagenomic ana­lysis revealed microbial interactions in anammox bacterial enrichment cultures: 1) NO_2_^–^ and/or NO_3_^–^ reduction by heterotrophs to supply NH_4_^+^ and/or NO_2_^–^ to anammox bacteria, and 2) the vitamin and amino acid auxotrophy of coexisting heterotrophs (Lawson *et al.*, 2017). However, (meta)genomic information on the microbes cooccurring with anammox bacteria remains limited, and metagenomic ana­lyses have investigated potential interactions between anammox bacteria and cooccurring bacteria (Speth *et al.*, 2016; Lawson *et al.*, 2017). The authors dedicated >10 years to the cultivation of phylogenetically different anammox bacteria, and obtained enrichment cultures of *B. sinica* (Oshiki *et al.*, 2011), *B. sapporoensis* (Narita *et al.*, 2017), *J. caeni* (Hira *et al.*, 2012; Ali *et al.*, 2015), *K. stuttgartiensis* (Oshiki *et al.*, 2018), and *Scalindua* sp. husus a7 (Kindaichi *et al.*, 2011). To the best of our knowledge, no other laboratory has maintained these phylogenetically diverse anammox bacterial enrichment cultures in parallel, and these cultures provide an excellent opportunity to examine the metabolic potential of anammox bacteria and cooccurring bacteria in a metagenomic ana­lysis.

Therefore, a metagenomic ana­lysis of these 5 anammox bacterial enrichment cultures was performed in the present study to retrieve the whole genome sequences of anammox bacteria and cooccurring bacteria and examine potential microbial interactions occurring in the enrichment cultures. Anammox bacterial cultures were maintained in membrane bioreactors (MBRs) as previously described (Oshiki *et al.*, 2013; Zhang and Okabe, 2017). Inorganic basal media fed into the MBRs contained KH_2_PO_4_ (24.4‍ ‍mg L^–1^), MgSO_4_·7H_2_O (60‍ ‍mg L^–1^), CaCl_2_ (51‍ ‍mg L^–1^), and 0.5‍ ‍mL of trace element solutions I and II (van de Graaf *et al.*, 1996). Equimolar amounts of NH_4_(SO_4_)_2_ and NaNO_2_ were supplemented into inorganic basal media at 5 to 10‍ ‍mM, and nitrogen loading rates were in the range of 0.1 to 0.6‍ ‍kg‍ ‍N‍ ‍m^–3^‍ ‍d^–1^. In cultivations of *K. stuttgartiensis* and *Scalindua* sp. husus a7, the artificial sea salt SEALIFE (Marine Tech) (Kindaichi *et al.*, 2011) was supplemented into inorganic basal media at final concentrations of 10 and 28‍ ‍g L^–1^, respectively. Anammox bacteria proliferated in the form of planktonic cells, which were harvested by centrifugation at 13,420×*g* for 10‍ ‍min for genomic DNA extraction. Total genomic DNA was extracted using the DNeasy Blood and Tissue kit (Qiagen) and then subjected to shotgun sequence library construction using the KAPA Hyper Prep Kit (for Illumina) (KAPA Biosystems) as described in Hirai *et al.* (2017). Between 3.4 and 5.7 M of 300-‍bp paired-end reads (corresponding to 1.0–1.7 Gb) were obtained for samples on the Illumina MiSeq sequencer. Reads were then subjected to adapter trimming using Trimmomatic version 0.36 (Bolger *et al.*, 2014), *de novo* assembly by the CLC Genomics workbench (word size 64, bubble size 500), and binning of the assembled contigs using MyCC (Lin and Liao, 2016). Gene annotation and completeness checks of the metagenome-assembled genomes (MAGs) obtained were performed using the DDBJ Fast Annotation and Submission tool (DFAST) (Tanizawa *et al.*, 2018). Average nucleotide identity (ANI) values were calculated in the DFAST pipeline and also using the OrthoANI tool (Lee *et al.*, 2016). Fourteen high-quality MAGs with >83% and <15.7% of completeness and contamination, respectively, were obtained (Table 1). The taxonomy of these MAGs was examined using GTDB-Tk v1.7.0 with release 202 data (Chaumeil *et al.*, 2019) and AnnoTree (Mendler *et al.*, 2019). Five MAGs were affiliated to 4 anammox bacterial genera, *Brocadia*, *Jettenia*, *Kuenenia*, and *Scalindua* (Fig. S1a), while other non-anammox bacterial MAGs were affiliated to the phyla *Planctomycetota* (Fig. S1a), *Bacteroidota* (Fig. S1b), and *Chloroflexota* (Fig. S1c). Anammox bacterial MAGs were identified as genomes of *B. sinica* (the HBSIN01 MAG), *B. sapporoensis* (the HBSAPP01 MAG), *J. caeni* (the JETCAE04 MAG), *K. stuttgartiensis* (the HKUEN01 MAG), and *Scalindua* sp. (the SCALA701 MAG) because they had >99.4% ANI to reference anammox bacterial genomes (Table S1). The relative abundance of anammox bacterial MAGs in metagenomic sequencing data was calculated by dividing the read numbers assigned to anammox bacterial MAG by total read numbers, which were generally high (37–64%), except for the* J. caeni* and *K. stuttgartiensis* biomasses (17 and 9%, respectively) (Table 1). Apart from anammox bacterial MAGs, *Chloroflexota* (JETCAE01, JETCAE02, and HKUEN02) and *Bacteroidota* (HBSIN02, HBSAPP04, JETCAE03, and SCALA702) MAGs were obtained from anammox bacterial enrichment cultures. A previous metagenomic sequencing ana­lysis of anammox bacterial enrichment cultures also retrieved *Chloroflexota* (Table S2) and *Bacteroidota* (Table S3) MAGs (Speth *et al.*, 2016; Bhattacharjee *et al.*, 2017; Lawson *et al.*, 2017; Mardanov *et al.*, 2019; Okubo *et al.*, 2021): *e.g.*, *Chloroflexota* MAGs (JETCAE01 MAG) and *Bacteroidota* MAGs (JETCAE03 MAG and HBSAPP04 MAGs) obtained in the present study showed high ANI values with *Anaerolineae* and *Ignavibacteria* MAGs obtained from anammox bioreactors operated by other research groups (Zhao *et al.*, 2019; Ali *et al.*, 2020) (Table S1). This result implies that anammox bioreactors fed with inorganic media containing NH_4_^+^ and NO_2_^–^ enrich phylogenetically-defined bacterial members as a core microbiome, as previously suggested by Lawson *et al.* (2017).

The metabolic capabilities of MAGs for central nitrogen and carbon metabolism were examined by performing a blastKOALA search using the KEGG database (Kanehisa *et al.*, 2016), and search hits were visualized using the KEGG Decoder (Graham *et al.*, 2018). Known anammox bacterial genomes commonly harbor the gene sets required for the anammox process (nitrite reduction, hydrazine synthesis, and hydrazine oxidation) and CO_2_ fixation via the Wood-Ljungdahl pathway (Strous *et al.*, 2006; Oshiki *et al.*, 2015, 2017) (Table S4). These gene sets are generally conserved on the anammox bacterial MAGs obtained. The nitrite reductase (Nir) of anammox bacteria is still controversial because the gene encoding a canonical Nir (cytochrome *cd_1_*-containing NirS and copper-containing NirK) is often missing in *Brocadia* genomes (Oshiki *et al.*, 2016; Okubo *et al.*, 2021), and neither *nirS* nor *nirK* was found in HBSIN01 and HBSAPP01 MAGs (Table S4). The involvement of atypical hydroxylamine dehydrogenase (rHao) in anammox bacterial nitrite reduction has been proposed (Kartal *et al.*, 2013; Oshiki *et al.*, 2016), and rHao was recently purified and characterized from a *K. stuttgartiensis* culture (Ferousi *et al.*, 2021). rHao lacks the tyrosine residue required for the crosslinking of catalytic haem 4 in Hao, and the gene encoding putative rHao was conserved among the anammox bacterial MAGs obtained in this study (Table S4). In addition, the SCALA701 MAG differed from the other known *Scalindua* genomes as follows: 1) SCALA701 MAG has *nirK* instead of *Scalindua nirS* (van de Vossenberg *et al.*, 2013; Oshiki *et al.*, 2017), and 2) SCALA701 HzsB and HzsG are encoded in each CDS as well as the known *Brocadiaceae* genomes, whereas the fusion protein of HzsBG is encoded in the genome of *Scalindua profunda* (van de Vossenberg *et al.*, 2013). Functional difference(s) resulting from the presence of *nirK* and separated *hzsBG* remain unclear and, thus, warrant further study.

Non-anammox bacterial *Planctomycetota*, *Chloroflexota*, and *Bacteroidota* MAGs have the gene sets required for fermentation (substrate-level phosphorylation; such as glycolysis) (Fig. 1) and respiration (cytochrome *c* oxidase and dissimilatory NO_3_^–^ reduction), whereas the MAGS of known inorganic carbon fixation pathways are absent. JETCAE02 (*Chloroflexota*) MAG harbors some of the genes involved in the Wood-Ljungdahl pathway, whereas the genes encoding key enzymes, namely, formate dehydrogenase and formate-tetrahydrofolate ligase, are missing. These features suggest that non-anammox bacterial *Planctomycetota*, *Chloroflexota*, and *Bacteroidota* are heterotrophic bacteria, whereas inorganic basal media fed into the operated MBRs and the nutrients required for heterotrophic growth were not available in influents. Extracellular polymeric substances (EPS) (Ali *et al.*, 2018), soluble microbial products (SMP) (Tsushima *et al.*, 2007; Oshiki *et al.*, 2011), and/or cell debris derived from anammox bacteria may be nutrient sources for heterotrophs. Anammox bacteria produce large amounts of EPS mainly composed of proteins and polysaccharides (Hou *et al.*, 2015; Jia *et al.*, 2017; Ali *et al.*, 2018), and the anammox bacterial MAGs obtained have the genes encoding the bacterial type II secretion system involved in protein secretion (GspDEFGIK) and the ABC transporters of lipopolysaccharide (LptBFG) and lipoprotein (LolCDE) involved in the formation of the lipopolysaccharide layer. *Chloroflexota* and *Bacteroidota* MAGs have the genes required for the degradation of polysaccharide chains (alpha-amylase and beta-glucosidase) (Fig. 1), indicating their metabolic potential for the degradation of EPS. *Chloroflexota* bacteria belonging to the class *Anaerolineae* are obligately anaerobic bacteria (Yamada and Sekiguchi, 2009; Nunoura *et al.*, 2013), and utilize a number of organic compounds, including sugars, with the production of short fatty acids and hydrogen gas (Sun *et al.*, 2016). *Chloroflexota* bacteria in an anammox bioreactor assimilate sucrose, glucose, and N-acetyl-glucosamine, as confirmed by microautoradiography and fluorescence *in situ* hybridization (Kindaichi *et al.*, 2012). The *Chloroflexota* MAGs obtained had genes encoding the thiamin transporter, but generally lacked the gene set required for thiamine biosynthesis (Fig. 1); *i.e.*, *thiFGHI* required for the synthesis of 4-methyl-5-(β-hydroxyethyl) thiazole phosphate, *thiCD* for the synthesis of 4-amino-5-hydroxymethyl-2-methylpyrimidine pyrophosphate, *thiE* for the synthesis of thiamine monophosphate, and *thiL* for the synthesis of thiamine pyrophosphate (Leonardi and Roach, 2004). On the other hand, *Planctomycetota* MAGs exhibited metabolic potential for thiamine synthesis, and these bacteria may supply thiamine to *Chloroflexota* bacteria in anammox bacterial enrichment cultures. The exchange of amino acids and vitamins between anammox bacteria and cooccurring bacteria was predicted based on the findings of previous metagenomic and metatranscriptomic ana­lyses (Lawson *et al.*, 2017), and the present results are consistent with this hypothesis. Among *Bacteroidota* bacteria, those belonging to the genera *Melioribacter* and *Ignavibacteria* are facultative anaerobic heterotrophs (Iino *et al.*, 2010; Podosokorskaya *et al.*, 2013), and may scavenge contaminated O_2_ in anammox bioreactors. *Melioribacter roseus* utilized a number of carbon compounds for fermentation, and proliferates with the production of acetate and H_2_ gas or by respiration using oxygen or NO_2_^–^ as an electron acceptor (Podosokorskaya *et al.*, 2013). In the present study, *Chloroflexota* and *Bacteroidota* MAGs harbored the genes required for dissimilatory NO_3_^–^ reduction to NO_2_^–^ or dissimilatory NO_2_^–^ reduction to NH_4_^+^ (DNRA) (Fig. 1). Anammox bacteria oxidize NO_2_^–^ to NO_3_^–^ to gain the reducing power for CO_2_ fixation (Kartal *et al.*, 2013 and references therein), and NO_3_^–^ concentrations are generally at >1‍ ‍mM in the operated MBRs. On the other hand, NH_4_^+^ and NO_2_^–^ are consumed by anammox bacteria in the MBRs, and may be a limiting substrate(s) of anammox bacteria after their depletion. Therefore, the production of NO_2_^–^ and/or NH_4_^+^ by *Chloroflexota* and *Bacteroidota* bacteria is beneficial for anammox bacteria. These interactions via NO_x_^–^ in the anammox bacterial community were proposed in previous metagenomic studies (Speth *et al.*, 2016; Lawson *et al.*, 2017), and the metabolic potential of the *Chloroflexota* and *Bacteroidota* MAGs obtained further rationalize this hypothesis.

In summary, the present study provides metagenome sequencing data obtained from 5 phylogenetically different anammox bacterial enrichment cultures in addition to genomic information on 14 high-quality MAGs. Anammox bacteria appear to supply organic matter (in the form of EPS, soluble microbial products, and cell debris), vitamins, and NO_3_^–^ to cooccurring heterotrophic bacteria. Cooccurring heterotrophic bacteria may scavenge contaminated O_2_ and prevent the accumulation of organic matter, which suppresses anammox activity (Tsushima *et al.*, 2007). Although the verification of microbial interactions by a culture-dependent ana­lysis is warranted (Murakami *et al.*, 2022), the genome data obtained supports previously proposed microbial interactions between anammox bacteria and cooccurring bacteria (Lawson *et al.*, 2017) and will advance our understanding of microbial interactions in anammox enrichment cultures. The clarification of these microbial interactions will provide insights into the specific reason(s) for unsuccessful isolation attempts of anammox bacteria, and metatranscriptomic and metaproteomic ana­lyses (Masuda *et al.*, 2017) in addition to the isolation of cooccurring bacteria are required to reveal microbial interactions in anammox bacterial communities.

## Data availability

Raw metagenomic sequence data obtained in the present study are available in the DDBJ nucleotide sequence database under the accession number DRA013237. The 14 assembled and annotated MAGs are deposited in the DDBJ nucleotide sequence database with the accession numbers shown in Table S5.

## Citation

Oshiki, M., Takaki, Y., Hirai, M., Nunoura, T., Kamigaito, A., and Okabe, S. (2022) Metagenomic Analysis of Five Phylogenetically Distant Anammox Bacterial Enrichment Cultures. *Microbes Environ ***37**: ME22017.

https://doi.org/10.1264/jsme2.ME22017

## Supplementary Material

Supplementary Material

## Figures and Tables

**Fig. 1.**
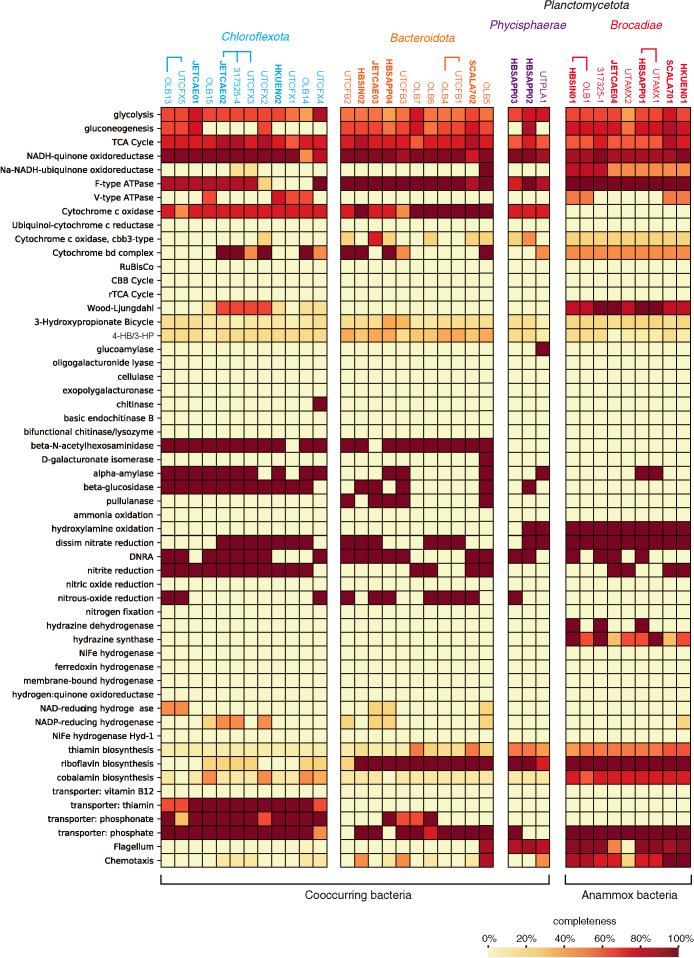
Metabolic potential of metagenome-assembled genomes (MAGs) obtained from anammox bacterial enrichment cultures. The figure includes MAGs in the present study (*i.e.*, HBSIN, HBSAPP, JETCAE, HKUEN, and SCALA7 MAGs) and those obtained from a partial-nitritation anammox reactor (UTPRO, UTCFX, UTCFB, UTPLA, and UTAMX MAGs), a sequencing batch anammox reactor (OLB MAGs), and an up-flow column anammox reactor (the 317325 MAGs). MAGs with a parenthesis share >97% of the average nucleotide identity values. The heatmap indicates metabolic pathway completeness calculated using the KEGG Decoder. The taxonomic affiliations of MAGs are available at the top of the heatmap.

**Table 1.** Metagenome-assembled genomes (MAGs) obtained from 5 anammox bacterial cultures. Taxonomic assignments were examined using GTDB-Tk and shown in Fig. S1.

Biomass^1)^	MAGs	Taxonomy^2)^	Total length	Contigs	GC	CDS	rRNA	tRNA	Completeness	Contamination	Abundance^3)^
BS	HBSIN01	*Planctomycetota*	3,980,744	87	42.4%	3,604	16S-1, 23S-1, 5S-1	48	96%	0.0%	64%
	HBSIN02	*Bacteroidota*	3,004,117	111	55.4%	2,565	16S-1, 23S-1, 5S-1	46	100%	0.0%	8%
BA	HBSAPP01	*Planctomycetota*	3,345,265	139	42.4%	2,758	16S-1, 23S-1, 5S-1	47	96%	0.0%	37%
	HBSAPP02	*Planctomycetota*	3,888,461	24	63.4%	3,148	16S-1, 23S-1, 5S-1	49	89%	0.8%	33%
	HBSAPP03	*Planctomycetota*	3,538,919	30	67.9%	3,045	16S-1, 23S-1, 5S-1	55	83%	1.2%	10%
	HBSAPP04	*Bacteroidota*	4,378,747	1118	47.1%	2,829	5S-1	44	100%	7.5%	4%
JC	JETCAE01	*Chloroflexota*	4,077,411	398	53.2%	3,598	23S-1, 5S-1	42	90%	8.3%	6%
	JETCAE02	*Chloroflexota*	3,195,621	90	60.9%	2,914	16S-1, 23S-1, 5S-1	45	91%	2.4%	9%
	JETCAE03	*Bacteroidota*	4,208,711	87	34.5%	3,744	16S-1, 23S-1, 5S-1	81	83%	15.7%	9%
	JETCAE04	*Planctomycetota*	3,935,265	95	40.0%	3,368	16S-1, 23S-1, 5S-1	46	96%	0.0%	17%
KS	HKUEN01	*Planctomycetota*	4,181,252	391	40.8%	3,539	16S-1, 23S-1, 5S-1	51	93%	12.5%	9%
	HKUEN02	*Chloroflexota*	2,777,596	539	52.8%	2,278	16S-1, 5S-1	43	100%	3.5%	3%
SC	SCALA701	*Planctomycetota*	4,498,465	120	41.1%	3,748	16S-1, 23S-1, 5S-1	43	96%	8.3%	52%
	SCALA702	*Bacteroidota*	4,901,315	164	38.8%	3,866	16S-1, 23S-1, 5S-1	42	100%	0.0%	12%

1) BS, BA, JC, KS, and SC correspond to cultures of *Brocadia sinica*, *Brocadia sapporoensis*, *Jettenia caeni*, *Kuenenia stuttgartiensis*, and *Scalindua* sp. husus a7, respectively. 2) Phylogenetic trees are available in Fig. S1. The closest reference genome and ANI scores are available in Table S1. 3) Relative abundance of the number of sequence reads assigned to each MAG to the total number of sequence reads.
