# Supplementary material for: Metagenomic Analysis of Five Phylogenetically Distant Anammox Bacterial Enrichment Cultures
Source: Microbes Environ. 2022 Jul 9;37(3):ME22017. doi: 10.1264/jsme2.ME22017 (PMC9530715; doi:10.1264/jsme2.ME22017)
Supplement: Supplementary file 1 — Supplementary Material [file 37_22017_s1.pdf]

Supplementary material

Metagenomic analysis of five phylogenetically-distant anammox bacterial culture collection

Mamoru Oshiki, Yoshihiro Takaki, Takuro Nunoura, Atsushi Kamigaito, and Satoshi Okabe

The supplementary material is available as a separated file. The file contains 1 supplementary figure and 5 supplementary tables.

Table S1. Reference genomes with the highest similarity with the MAGs obtained in the present study. Reference genomes were shown as the GenBank Assembly accession numbers. Values with parenthesis indicate ANI scores. NA: not available because a reference genome was not assigned on the GtDB-tk v1.7.0 search using a reference genome data set release 202.

MAGs	Taxonomy	Closest reference
HBSIN01	*Planctomycetota*	GCF_000949635.1 (99.99)
HBSIN02	*Bacteroidota*	NA
HBSAPP01	*Planctomycetota*	GCF_001753675.2 (99.79)
HBSAPP02	*Planctomycetota*	GCA_003576875.1 (99.89)
HBSAPP03	*Planctomycetota*	NA
HBSAPP04	*Bacteroidota*	GCA_008363265.1 (98.61)
JETCAE01	*Chloroflexota*	GCA_008363285.1 (98.1)
JETCAE02	*Chloroflexota*	GCA_002473085.1 (98.25)
JETCAE03	*Bacteroidota*	GCA_013285405.1 (99.84)
JETCAE04	*Planctomycetota*	GCF_000296795.1 (99.99)
HKUEN01	*Planctomycetota*	GCF_900232105.1 (99.4)
HKUEN02	*Chloroflexota*	NA
SCALA701	*Planctomycetota*	GCA_004282745.1 (99.99)
SCALA702	*Bacteroidota*	NA

Table S2 Average nucleotide identity (ANI) values of the *Chloroflexota* bins obtained from anammox bacterial cultures: OLB14 (LMZR00000000), OLB15(LMZS00000000), UTCFX5 (MWSX00000000), UTCFX1 (MWTB00000000), UTCFX2 (MWTA00000000), 317325-4 (BLAA01000001- BLAA01000004), OLB13 (JZRA00000000), JETCAE01, JETCAE02, and HKUEN02 (this study). The ANI values in the table were calculated using the OrthoANI tool.

	OLB14	JETCAE01	UTCFX3	UTCFX4	OLB15	UTCFX5	JETCAE02	HKUEN02	UTCFX1	UTCFX2	317325-4	OLB13
OLB14	100%	68.9%	65.8%	63.6%	62.5%	62.7%	66.2%	68.7%	68.4%	63.6%	66.8%	62.8%
JETCAE01		100%	71.6%	64.8%	64.3%	64.1%	71.2%	70.4%	69.8%	66.2%	72.7%	64.6%
UTCFX3			100%	64.6%	64.1%	65.4%	98.0%	70.2%	70.1%	67.5%	98.5%	65.4%
UTCFX4				100%	64.0%	64.3%	64.5%	65.6%	64.9%	63.8%	65.2%	64.3%
OLB15					100%	66.9%	64.7%	64.4%	63.8%	63.7%	65.0%	67.1%
UTCFX5						100%	65.8%	64.3%	63.7%	64.8%	66.0%	99.3%
JETCAE02							100%	70.7%	70.4%	67.8%	97.9%	66.2%
HKUEN02								100%	92.6%	65.8%	70.9%	64.2%
UTCFX1									100%	65.6%	70.4%	64.1%
UTCFX2										100%	67.6%	70.0%
317325-4											100%	66.0%
OLB13												100%

Table S3 Average nucleotide identity (ANI) values of the *Bacteroidota* bins obtained from anammox bacterial cultures:, UTCHB1 (MWSW00000000), UTCHB2 (MWSV00000000), UTCHB3 (MWSU00000000), OLB4 (JYPE00000000), OLB6 (LLZP00000000), OLB7 (LMYZ00000000), OLB5 (LLZO00000000), HBSAPP04, HBSIN02, JETCAE03, and SCALA702 (this study). The ANI values in the table were calculated using the OrthoANI tool.

	UTCHB1	UTCHB2	UTCHB3	HBSAPP04	HBSIN02	JETCAE03	SCALA702	OLB4	OLB6	OLB7	OLB5
UTCHB1	100%	65.0%	64.0%	64.0%	63.6%	64.9%	65.2%	100.0%	64.1%	63.2%	65.9%
UTCHB2		100%	65.6%	65.9%	62.4%	71.2%	66.8%	65.4%	62.9%	61.7%	66.2%
UTCHB3			100%	70.9%	62.8%	65.9%	65.0%	63.9%	62.6%	62.4%	64.6%
HBSAPP04				100%	64.6%	65.9%	65.0%	64.5%	64.5%	62.8%	64.6%
HBSIN02					100%	63.7%	63.6%	63.6%	63.7%	64.9%	63.7%
JETCAE03						100%	66.4%	65.4%	62.7%	63.0%	66.2%
SCALA702							100%	65.7%	63.8%	62.8%	65.0%
OLB4								100%	64.7%	63.2%	66.5%
OLB6									100%	64.1%	63.8%
OLB7										100%	63.8%
OLB5											100%

Table S4 Core genes required for anammox process and the Wood-Ljungdahl pathway; n.f; not found (*e*-value; $>10^{-15}$).

Gene	Product	Query	HBSIN01	HBSAPP01	JETCAE04	HKUEN01	SCALA701
<u>Nitrite reduction</u>							
*nir*S	*cd_1* nitrite reductase	SCALIN_C43_0069	n.f	n.f	n.f	HKUEST01_03400	n.f
nirK	*copper-containing nitrite reductase*	KSU1_D0929	n.f	n.f	JETCAE04_07930	n.f	SCALA7_22740
rHao	*hydroxylamine dehydrogenase*	kustc0458	HBSIN01_18650	HBSAPP01_22900	JETCAE04_05620	HKUEST01_18850	SCALA7_07650
<u>Hydrazine synthesis</u>							
*hzs*B	hydrazine synthase beta subunit	BROSI_A2674	HBSIN01_35990	HBSAPP01_27400	JETCAE04_09970	HKUEST01_27330	SCALA7_20470
hzsG	hydrazine synthase gamma subunit	BROSI_A2675		HBSAPP01_27390	JETCAE04_09960	HKUEST01_27340	SCALA7_20460
*hzs*A	hydrazine synthase alpha subunit	BROSI_A2676	HBSIN01_35330	HBSAPP01_27380	JETCAE04_09980	HKUEST01_27350	SCALA7_20450
<u>Hydrazine oxidation</u>							
hdh	hydrazine dehydrogenase	SCALIN_C22_0030	HBSIN01_18650	HBSAPP01_22900	JETCAE04_05620	HKUEST01_18850	SCALA7_07650
			HBSIN01_17020	HBSAPP01_02590	JETCAE04_24830	HKUEST01_30420	
<u>Wood-Ljungdahl pathway (CO_2 fixation)</u>							
*fdh*A	formate dehydrogenase	SCALIN_C28_0320	HBSIN01_02630	HBSAPP01_21440	JETCAE04_22420	HKUEST01_00010	SCALA7_36800
			HBSIN01_29520	HBSAPP01_19300	JETCAE04_19020		SCALA7_28230

		HBSIN01_08260	HBSAPP01_10650	JETCAE04_20200		
*acs*A	SCALIN_C04_0234	HBSIN01_30750	HBSAPP01_07350	JETCAE04_28180	HKUEST01_00040	SCALA7_20870
		HBSIN01_09560			HKUEST01_30690	SCALA7_23730
*acs*B	SCALIN_C04_0235	HBSIN01_09570	HBSAPP01_07340	JETCAE04_28190	HKUEST01_00050	SCALA7_33880
						SCALA7_15100
*acs*C	SCALIN_C04_0237	HBSIN01_09590	HBSAPP01_07320	JETCAE04_28210	HKUEST01_00070	SCALA7_15120
*acs*D	SCALIN_C04_0240	HBSIN01_09620	HBSAPP01_07290	JETCAE04_28240	HKUEST01_00100	SCALA7_15150
*acs*E	SCALIN_C04_0241	HBSIN01_09630	HBSAPP01_07280	JETCAE04_28250	HKUEST01_00110	SCALA7_15160
*acs*F	SCALIN_C04_0239	HBSIN01_09610	HBSAPP01_07300	JETCAE04_28230	HKUEST01_00090	SCALA7_15140
		HBSIN01_09550	HBSAPP01_07360	JETCAE04_28170	HKUEST01_32350	SCALA7_15090
					HKUEST01_00030	

Table S5. DDBJ accession numbers of the metagenome-assembled genomes (MAGs) obtained in the present study.

MAGs	Accession numbers
HBSIN01	BQMK01000001-BQMK01000087
HBSIN02	BQML01000001-BQML01000111
HBSAPP01	BQMM01000001-BQMM01000139
HBSAPP02	BQMN01000001-BQMN01000024
HBSAPP03	BQMO01000001-BQMO01000030
HBSAPP04	BQMP01000001-BQMP01001118
JETCAE01	BQMQ01000001-BQMQ01000398
JETCAE02	BQMR01000001-BQMR01000090
JETCAE03	BQMS01000001-BQMS01000087
JETCAE04	BQMT01000001-BQMT01000095
HKUEN01	BQMU01000001-BQMU01000391
HKUEN02	BQMV01000001-BQMV01000539
SCALA701	BQMX01000001-BQMX01000120
SCALA702	BQMY01000001-BQMY01000164

Figure legends for supplementary figures.

Figure S1 Taxonomic assignments of the metagenome-assembled genomes (MAGs): a) *Planctomycetota*, **b)** *Chloroflexota*, and **c)** *Bacteroidota* MAGs. Phylogenetic trees were referred from those available in the AnnoTree software, and the tree was calculated using the GTDB release 202 data. The taxonomic positions where the MAGs were assigned are highlighted with blue color, and the origin and name of the MAGs are shown with symbols and IDs of MAGs. Abbreviations of the enrichment cultures are as following; KS for *Kuenenia stuttgartiensis*, BS for *Brocadia sinica*, BA for *Brocadia sapporoensis*, JC for *Jettenia caeni*, and SC for *Scalindua* sp. husus a7, respectively. The taxonomic positions with the symbol AMX (open square) contain the bacterial MAGs previously obtained from the anammox bioreactors operated in other studies. The numbers with parenthesis are the numbers of the genomes in the group. *; only 1 genome is deposited in the taxonomic group. **; no corresponding taxonomy was available, and a higher-level taxonomy is shown in the phylogenetic tree.

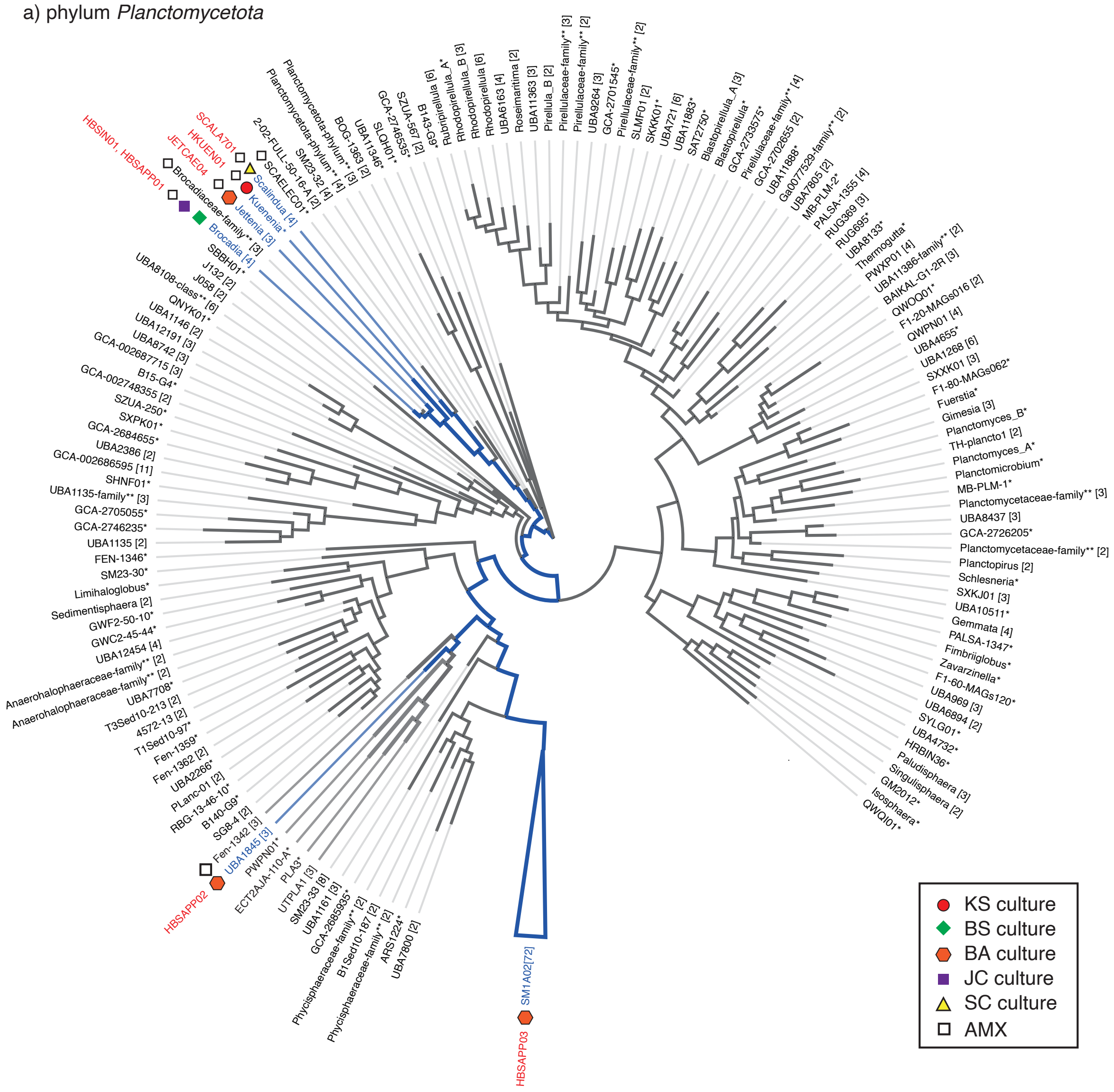

Fig. S1 (Oshiki et al.)

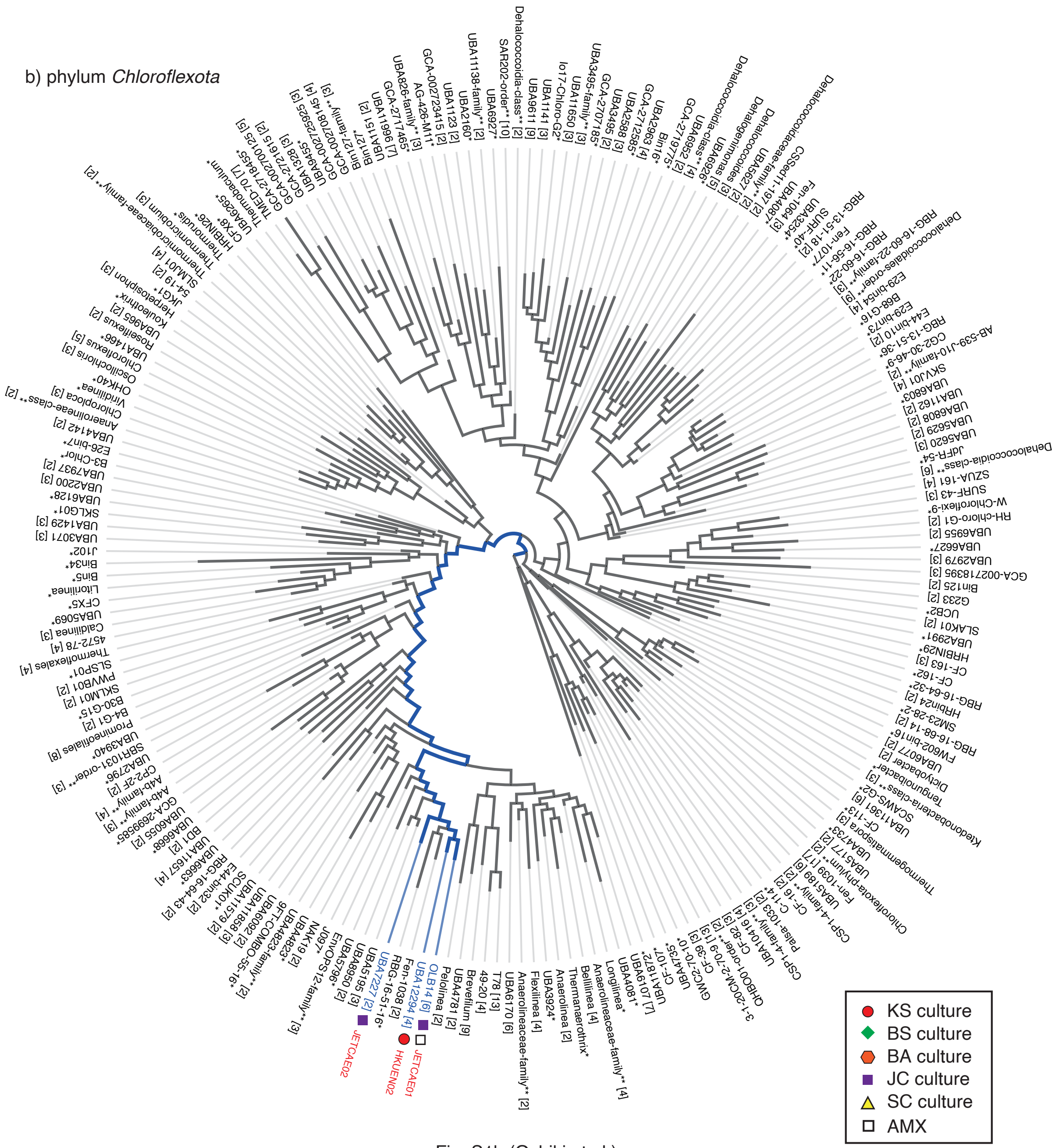

Fig. S1b (Oshiki et al.)

42

c) phylum *Bacteroidota*

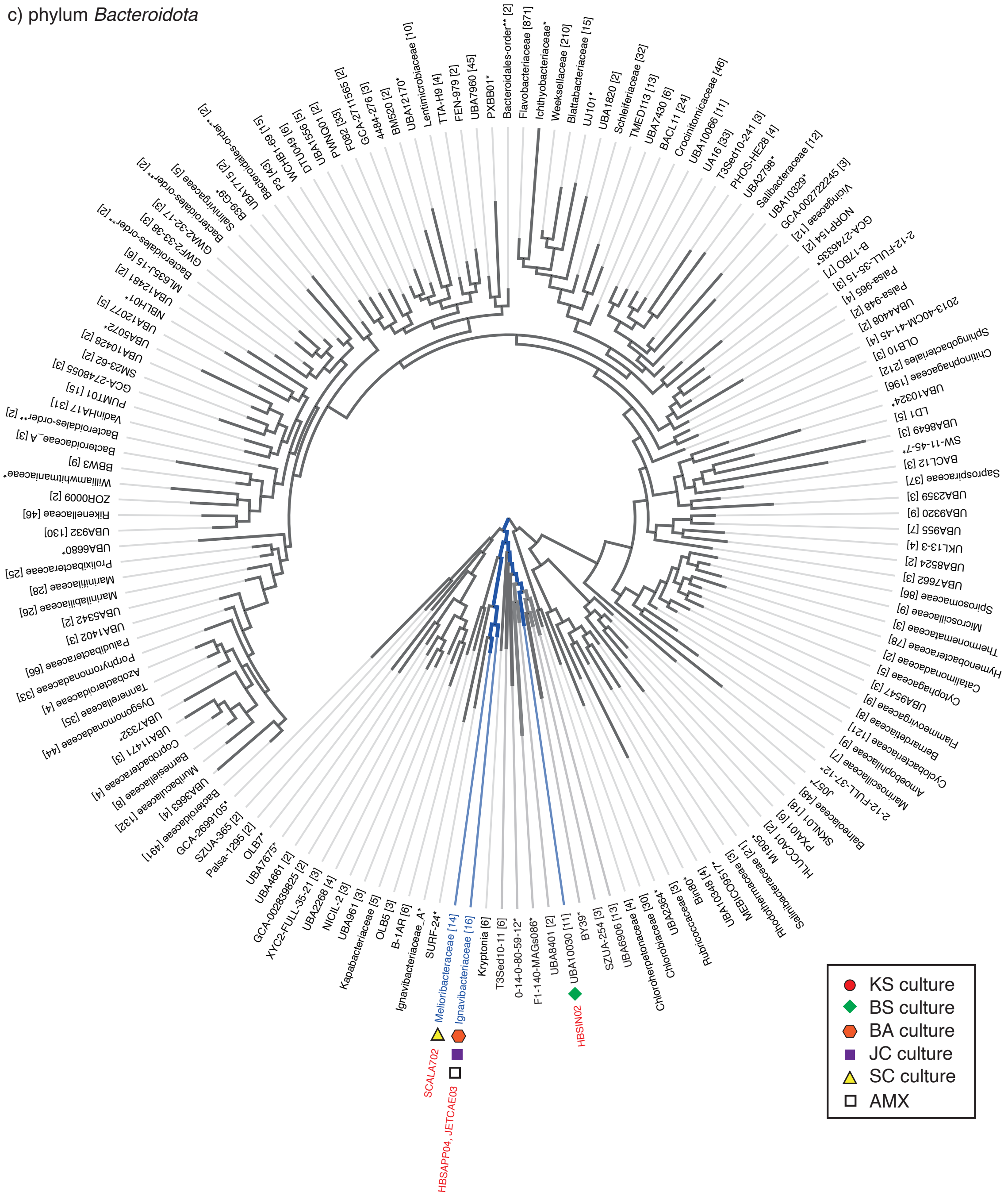

Fig. S1c (Oshiki et al.)

43
